# The approval of the first malaria vaccine: The beginning of the end of the malaria epidemic

**DOI:** 10.7189/jogh.12.03087

**Published:** 2022-12-23

**Authors:** Isaac Olufadewa, Deborah Akinrinde, Miracle Adesina, Ruth Oladele, Toluwase Ayorinde, Uvie Omo-Sowho

**Affiliations:** 1 Slum and Rural Health Initiative, Akobo, Nigeria; 2 College of Medicine, University of Ibadan, Ibadan, Nigeria; 3 Faculty of Veterinary Medicine, University of Ibadan, Ibadan, Nigeria

In the last 50 years, at least 34 million people have died from malaria, 96% of whom were from Africa [1]. Despite US$401 billion spent on the fight against malaria in the past decade [2] and numerous national malaria programmes and policies implemented, only modest gains have been recorded as malaria continues to drastically impact the health and livelihoods of people in sub-Saharan African countries due to weak health systems, inadequate political commitment, corruption, poverty, illiteracy, conflicts among others [2]. However, after several decades of developing the world’s first malaria vaccine (**Figure 1**), The World Health Organization’s (WHO) approval of the RTS,S/AS01 malaria vaccine for children between five and 17 months of age at first vaccination in October 2021 offered a ray of hope for eliminating malaria in Africa in the coming decades [3]. Here we explore some previous innovative vector control methods and their shortcomings in malaria control, the opportunities associated with the recently approved malaria vaccine, and areas of improvement for future malaria vaccines, while we also recommend strategies for the widespread adoption of the malaria vaccine.

**Figure 1:**
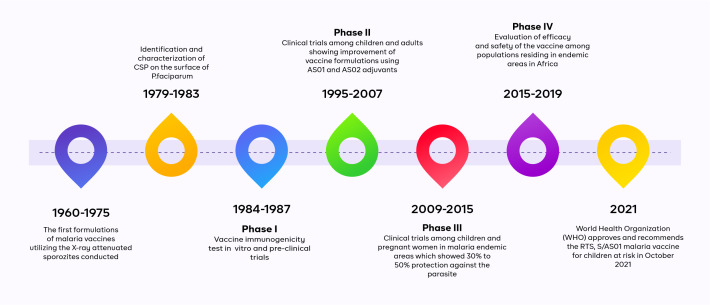
RTS,S malaria vaccine timeline.

## PREVIOUS EFFORTS

In previous decades, both modern/innovative and traditional insecticide-based vector control methods have been the most important tools for interrupting the malaria transmission cycle and have contributed to the alleviation of the burden of the malaria disease. Seventy-nine percent of the reduction in the number of malaria-related deaths between 2000 and 2015 was attributed to the widespread use of insecticide-treated nets and indoor residual spraying [4]. High-tech Aquatain-spraying anti-malaria drones (developed and first tested in Tanzania to help control vectors by larval source management) helped fight malaria by complementing the more labour-intensive method of manually identifying larval habitat from the ground [5]. The successes of these vector control methods face constant threats, as there has been a dramatic increase in the frequency and intensity of insecticide resistance in malaria vector populations [4] and the impracticality of the use of anti-malarial drones in rural areas with diffuse seasonal larval habitat [5].

Seasonal malaria chemoprevention for plasmodium falciparum malaria control, first advocated for use by the WHO in 2012 in highly seasonal transmission areas of the Sahel sub-region of Africa, has contributed to the reduction in the prevalence of malaria by causing a 75% protection against its uncomplicated and severe forms in children aged under five years [6]. Also, social and economic development has caused changes to housing conditions, contributing to an improvement in environmental hygiene and the reduction in the spread of malaria [1]. For instance, evidence from Demographic and Health Surveys conducted in 21 sub-Saharan countries between 2008 and 2015 found that the change from traditional housing to modern ones was associated with a 14% reduction in the prevalence of malaria [7].

## INTRODUCING MALARIA VACCINE AND EMERGING CONCERNS

The quest for a malaria vaccine started more than a century ago. Recently, a first-generation vaccine, RTS,S, based on a recombinant protein has received approval from the World Health Organization (WHO) and has been recommended for broader-scale use, with the potential for averting millions of malaria cases and hundreds of thousands of malaria-related deaths. The vaccine targets the sporozoite stage of the plasmodium falciparum lifecycle, preventing liver infection, where the parasite would otherwise mature, multiply, and infect the host red blood cells [8].

Phase 3 trials for the vaccine, which took place between 2009-2014 in Burkina Faso, Gabon, Ghana, Kenya, Malawi, Mozambique, and Tanzania, provided evidence that the vaccine prevented 39% of clinical malaria cases and 29% of severe malaria cases over four years of follow-up in children between the ages of five and 17 months of age who received three doses of the RTS,S vaccine together with a booster dose [9]. The vaccine has been found to have a strong safety profile from the evidence provided by the administration of over 2.3 million doses across three African countries, cost-effective in areas of moderate to high plasmodium falciparum transmission and feasible to deliver through routine immunization systems [10].

However, there are emerging concerns about the RTS,S vaccine’s long-term efficacy, warranting further research. A study of the RTS,S/AS01-induced anti-CSP IgG antibodies kinetics in young Kenyan children over a seven-year period identified long-lived, but declining efficacy of the vaccine [10]. Initial protection against clinical malaria was found in a group of young children who received three RTS,S vaccine doses. The endpoint after seven years of observation was, however, the resurgence of clinical malaria with evidence of negative efficacy in children [10].

## RECOMMENDATIONS

Now that a malaria vaccine has been approved, a continental strategy for deploying them in Africa must be developed, with the African Union and the regional bodies (such as COMESA and ECOWAS) playing a leading role, alongside other multilateral agencies. Countries can adopt this plan and further supplement and adapt it based on their context. They also must have procedures in place for national regulatory bodies to rigorously ascertain vaccine safety and efficacy and swiftly approve vaccines for use and be ready to carry out unprecedented mass vaccination. There must be coordination systems between national regulatory agencies and customs authorities; logistics and delivery systems must be carefully planned to maintain cold chain systems for monitoring and managing of adverse post-vaccination symptoms. Each country should also focus on supply chain and distribution strategy for the identification of target populations and a post-introduction evaluation must be done six to 12 months after the launch of the vaccine, as recommended by the WHO guideline for new vaccines. Countries must craft a vaccination component for their malaria intra-action review to enable continued review of vaccine deployment and close monitoring of vaccine rollout, thus informing decisions for addressing encountered obstacles.

We propose the establishment of a Malaria Vaccine Coalition which can be led by the WHO, Global Alliance for Vaccines and Immunization (GAVI), UNICEF, and the Global Fund, with national NGOs and local CBOs that can ensure the progress in the development of a better, multi-stage malaria vaccine, evaluate the cost-effectiveness, and promote equitable distribution of the licensed malaria vaccines to give affected countries access to the vaccines through the advance market commitment model. The model has been used to successfully distribute new vaccines in previous times in low- and middle-income countries. African countries should also consider covering the cost of the vaccine to make it free for children below five years, roll it out through the primary health care centres, and integrate it into their respective national routine immunization schemes.

Vaccines alone will not solve the problem of malaria – significant progress will be impossible without a well-trained, knowledgeable, and empowered cadre of health workers delivering it. Furthermore, there is a high possibility that the excitement over the recently approved vaccine will overshadow existing malaria prevention and control measures. The fact that a malaria vaccine exists does not mean that funders, governments, and multilateral organizations should neglect the currently available malaria control measures. Better collection and use of data to plan, implement, and track progress will enable more efficient use of available resources.

Challenges associated with the COVID-19 vaccine have taught us that public confidence in vaccination can be fragile. Malaria vaccination programs will only succeed if there is a widespread belief that the vaccine is safe, of optimum efficacy, and that there are policies and programmes to ensure its equitable distribution. Effective communication and public health advocacy that conveys trust are also important. Clinical trial results should be publicly available. Government agencies must work in synergy with civil society organizations to sensitise and educate community members to promote vaccine uptake. Civil society organizations have invaluable access to vulnerable populations in remote communities, urban slums, and other malaria-endemic areas, and have historically played significant roles in mobilizing public support. CSOs can complement government efforts to promote awareness in local communities and ultimately drive acceptance of the malaria vaccine. Traditional media and popular online social media platforms can play a key role in addressing mis/disinformation, stimulating public engagement, and promoting public acceptance of the malaria vaccine to increase vaccine coverage.

**Figure:**
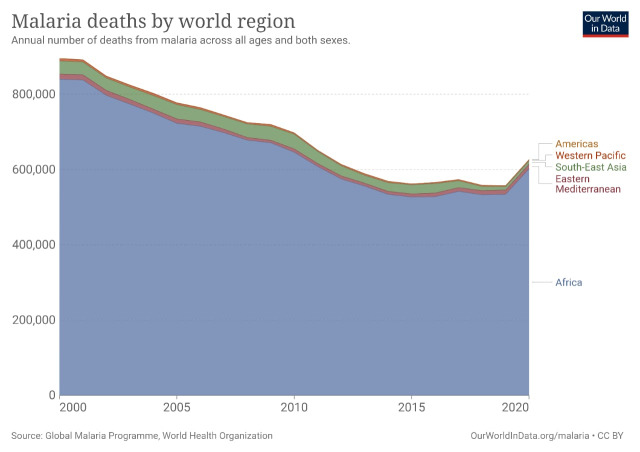
Photo: Malaria deaths by world region. Source: Global Malaria Programme, World Health Organization (World in Data, 2022). Used with permission.

The development and deployment of the recent malaria vaccine is an opportunity to strengthen the weak health systems in many sub-Saharan African countries. It calls for collective effort in ensuring capacity-building of the health workforce, a better coordination system, effective communication, public health advocacy, and marketing, which could be game-changing towards the eradication of needless childhood deaths from malaria. The malaria vaccine undoubtedly holds a promise of longevity to Africa’s beautiful children, productivity to her teeming youths, and possibilities to the continent. It gives us hope for a sub-Saharan Africa that is “malaria-free”.

## References

[1] Institute for Health Metrics and Evaluation. Global Burden of Disease Collaborative Network. Global Burden of Disease Study 2017 (GBD 2017) Results. Seattle, United States: Institute for Health Metrics and Evaluation (IHME), 2018. Available: http://ghdx.healthdata.org/gbd-results-tool. Accessed: 13 January 2022.

[2] Institute for Health Metrics and Evaluation. Financing Global health/Viz Hub: Flows of Development Assistance for Health. 2021. Available: https://vizhub.healthdata.org/fgh/%E2%80%8B. Accesssed: 14 January 2022.

[3] World Health Organization. WHO recommends ground breaking malaria vaccine for children at risk; Historic RTS, S/AS01 recommendation can reinvigorate the fight against malaria. 2021. Available: https://www.who.int/news/item/06-10-2021-who-recommends-groundbreaking-malaria-vaccine-for-children-at-risk. Accessed: 13 January 2022.

[4] Cibulskis RE Alonso P Aponte J Aregawi M Barrette A Bergeron L Malaria: Global progress 2000 - 2015 and future challenges. Infect Dis Poverty. 2016;5:61. 10.1186/s40249-016-0151-8 27282148 PMC4901420

[5] Stanton MC Kalonde P Zembere K Hoek Spaans R Jones CM The application of drones for mosquito larval habitat identification in rural environments: a practical approach for malaria control? Malar J. 2021;20:244. 10.1186/s12936-021-03759-2 34059053 PMC8165685

[6] Diawara F Steinhardt LC Mahamar A Traore T Kone DT Diawara H Measuring the impact of seasonal malaria chemoprevention as part of routine malaria control in Kita, Mali. Malar J. 2017;16:325. 10.1186/s12936-017-1974-x 28797263 PMC5553795

[7] Tusting LS Bottomley C Gibson H Kleinschmidt I Tatem AJ Lindsay SW Housing Improvements and Malaria Risk in Sub-Saharan Africa: A Multi-Country Analysis of Survey Data. PLoS Med. 2017;14:e1002234. 10.1371/journal.pmed.1002234 28222094 PMC5319641

[8] White MT Verity R Griffin JT Asante KP Owusu-Agyei S Greenwood B Immunogenicity of the RTS,S/AS01 malaria vaccine and implications for duration of vaccine efficacy: secondary analysis of data from a phase 3 randomised controlled trial. Lancet Infect Dis. 2015;15:1450-8. 10.1016/S1473-3099(15)00239-X 26342424 PMC4655306

[9] RTS,S Clinical Trials Partnership Agnandji ST Lell B Soulanoudjingar SS Fernandes JF Abossolo BP First results of phase 3 trial of RTS,S/AS01 malaria vaccine in African children. N Engl J Med. 2011;365:1863-75. 10.1056/NEJMoa1102287 22007715

[10] Olotu A Fegan G Wambua J Nyangweso G Leach A Lievens M Seven-Year Efficacy of RTS,S/AS01 Malaria Vaccine among Young African Children. N Engl J Med. 2016;374:2519-29. 10.1056/NEJMoa1515257 27355532 PMC4962898

